# A Study of Social Isolation, Multimorbidity and Multiple Role Demands Among Middle-Age Adults Based on the Canadian Longitudinal Study on Aging

**DOI:** 10.1177/00914150211040451

**Published:** 2021-10-12

**Authors:** Andrew V. Wister, Lun Li, Barbara A. Mitchell

**Affiliations:** 1 Department of Gerontology, Gerontology Research Centre, Simon Fraser University, Vancouver, Canada; 2 Gerontology Research Centre, 33507 Simon Fraser University, Vancouver, BC, Canada; 3 Department of Gerontology & Department of Sociology/Anthropology, 33507 Simon Fraser University, Burnaby, BC, Canada

**Keywords:** social isolation, multimorbidity, middle-life, multiple roles, caregiving, CLSA

## Abstract

Given the increasing complexity and fluidity of parenting, caregiving, and paid work patterns, in tandem with an increased risk of multimorbidity in mid-life, this study examines the relationship between these three concurrent roles and social isolation among middle-aged persons across multimorbidity statuses. Drawing upon life course theory, we applied linear mixed models to analyze 29,847 middle-aged participants from two waves of the Canadian Longitudinal Study on Aging. Findings reveal that participants experience greater social isolation over time, albeit the difference is extremely small. Among participants without multimorbidity, holding multiple roles is associated with lower social isolation. For those with multimorbidity, being employed full-time and providing intensive care are associated with social isolation. The occurrence of multiple roles demonstrates unique associations with social isolation among those with and without multimorbidity over time. Future research should study multimorbidity as a salient contextual variable. Moreover, enhanced support is needed for multimorbid middle-aged individuals with different role demands.

## Introduction

There is a growing and widespread interest in studying multiple role demands and transitions among the middle-aged generation given their increasing complexity, fluidity, and influence on human development (Lachman et al., 2015). The middle-aged generation typically refers to the segment of the population aged between 45 and 64 years old and constitutes about 28.3% of the Canadian population (Statistics Canada, 2019). Consequently, most of the middle-aged generation are members of the “sandwich generation,” who often assume multiple and competing generational, familial, and work roles, including parenthood, elder care, and paid employment (Chassin et al., 2010; Smith-Osborne & Felderhoff, 2014). Notably, the multiple roles assumed by the middle-aged generation are complicated by recent social, economic, and demographic trends, such as delayed childbearing, increased caregiving needs to support family members, more female labor market participation, and rapid population aging (Burke, 2017; Mitchell, 2021). Furthermore, this phase of the life course can have a prolonged and significant impact on the aging process itself in later life in terms of health and well-being outcomes (Chassin et al., 2010; Lachman & Agrigoroaei, 2010).

Research into the relationship between multiple roles and social, health, or wellbeing outcomes has produced substantial evidence supporting both positive and negative consequences, yet several gaps in this research remain (DeRigne & Ferrante, 2012; Seeman et al., 2011; Sinha, 2017). One area of relevance is the influence of experiencing competing multiple roles on social isolation over time among the middle-aged generation. Additionally, since middle-age is the period in life at which time chronic illness onset occurs, a question arises with respect to the association between juggling and competing role demands and patterns of social isolation interacting with experiences of multimorbidity. This study aims to understand these interrelated processes of aging by utilizing longitudinal data that allows for modeling and understanding change in mid-life experiences.

## Understanding Multiple Roles Through the Life Course

Life-course theory has been applied to the nexus of multiple work–family roles and health-related transitions over the various developmental phases of adulthood (Demerouti et al., 2012; Eifert et al., 2016). This school of thought has helped to elucidate the consequences and continuity of changing roles and transition statuses over the family life course (Halfon & Hochstein, 2002; Moen & Wethington, 1999). Elder and colleagues (Elder, 1998; Elder et al., 2003) also noted the salience of historical time and place; diversity in family life, the interdependency of “linked lives” in shaping interlocking roles and relationships, and the long-term cumulative consequences of earlier life course experiences and transitions.

During middle-age, individuals tend to undertake several interrelated social and familial roles, such as working, parenting, and caregiving (Moen & Wethington, 1999). Adoption of these roles can have profound influence on the lives of individuals traversing the middle life course. Notably, multiple role demands can profoundly affect the psychological and physical health of adult-child caregivers according to a study based on seven waves of the U.S. Health and Retirement Study (Barnett, 2015). Another potential social impact is social isolation, which is closely related to the social and interpersonal relationships and interactions connected to mid-life transitions (Kramer & Lambert, 1999). Indeed, Hansen and Slagsvold (2015) contended that the combination of intensive caregiving and reductions in paid labor force attachment, which are often linked, yield the highest burden on family caregivers, including loneliness and diminished social engagement.

Furthermore, the life course is highly gendered in terms of potential interference with one's social life due to the high demands and the interdependence of work and family roles (Lin & Burgard, 2018). For example, researchers reveal that significant differences related to working and parenting pressures exist across the life course stages and they tend to disadvantage women (Lin & Burgard, 2018). The middle-aged period is a particularly critical time in the life course because it is the time individuals, especially women, tend to also provide elder care, while also needing to prepare for retirement financially through stable employment (Pavalko & Artis, 1997). Moreover, in Canada, it is estimated that women enter their first elder care roles around the age of 46 years old, whereas it is 48 years old for men (Fast et al., 2013). Overall, life course theory is valuable for framing how and why multiple roles might differentially affect levels of social isolation among men and women in mid-life.

## Social Isolation and Multimorbidity Among the Middle-Aged Generation

Social isolation has been widely studied as a public health concern but often with different definitions and indicators (Gilmour & Ramage-Morin, 2020; Holt-Lunstad, 2017). In the current study, we follow recent developments of a comprehensive conceptualization of social isolation, which integrates the objective structural and functional dimensions of social relationship, as well as the subjective dimension of perceived social relationship (Valtorta, Kanaan, Gilbody and Hanratty, 2016; Wister et al., 2019). Specifically, this study defines social isolation as objective isolation, featured with low social participation, limited social interaction and network, in tandem with subjective isolation, including perceptions of loneliness and unsatisfied assessment of social relationships.

Low to moderate levels of social isolation have been reported among middle-aged population due to varying measures and populations. A recent study based upon a Canadian population revealed that about 5% of middle-aged Canadians are socially isolated using an index based on five domains, and around 10% of them are lonely (Menec et al., 2019). In addition, based on the Swiss Health Survey, roughly 34% of middle-aged individuals reported feelings of loneliness sometimes to very often, and about 23% of them were at least partly socially isolated (Hämmig, 2019). Also, at least 26% of participants aged between 45 and 59 years old were socially isolated in a national population-based study in China (Zhou et al., 2019). However, the documentation of social isolation among the middle-aged generation remains scarce, mainly because social isolation is less of a concern among this group relative to the older aged population (Chang et al., 2015).

A vast literature documenting the damaging consequences of social isolation on people's health and wellbeing is based on studies with older populations or a mix of middle-aged and older adults. A higher level of social isolation is associated with increasing risk of psychological difficulties (e.g., depression, anxiety), decreased cognitive function, worse physical health (e.g., increased blood pressure, coronary heart disease), even higher mortality (Beller & Wagner, 2018; Holt-Lunstad, 2017; Keefe et al., 2006; Menec et al., 2019; Valtorta, Kanaan, Gilbody, Ronzi, et al., 2016; Wister et al., 2019). Social isolation is detrimental to health and wellbeing due to its deleterious effect on people's resilient to stress, immune functioning, and the ability to perform healthy behaviors, to name a few (Beller & Wagner, 2018; Smith-Osborne & Felderhoff, 2014).

Although sparse, studies based solely on middle-aged participants also suggest that social isolation poses many health risks. For instance, Windsor et al. (2016) reported that middle-aged participants embedded in more diverse social networks have also been found to report better mental health than those who had a restricted network. Also, a significant association between lack of social support and the incidence of coronary heart disease has been supported (Grant et al., 2009). Additionally, evidence shows that individuals with supportive social relationship during mid-life have better functional health or cognitive capabilities in later life (Lachman et al., 2015; Seeman et al., 2011). Therefore, the concurrent and longitudinal association between social isolation and deleterious health and wellbeing underscores the importance and need for the study of social isolation among the middle-aged generation.

There is a trend of early onset of multimorbidity among general population. Multimorbidity usually refers to the occurrence of two or more chronic conditions, and it leads to reduced functional status, increased rate of disability and mortality, and poorer health-related quality of life (Barnett et al., 2012; Walker et al., 2016). Although the prevalence of multimorbidity increases with age, multimorbidity has become common in mid-life in many countries (Roberts et al., 2015; Sakib et al., 2019). Specifically, Sakib et al. (2019) reported increasing multimorbidity (using 3+ concurrent conditions) by age – 30% among Canadians aged between 45 and 49 years old; 34% for 50–54 years old; 45% for 55–59 years old; and 52% for those aged 60–64 years old. Roberts et al. (2015) contended that the odds of being multimorbid are higher among low-income middle-aged Canadians than for the general older population.

Overall, few studies have incorporated a focus on multimorbidity with multiple roles in mid-life; yet one would anticipate that their interaction would affect social connectedness. One known study based on the aging population in Europe (Schmidt et al., 2016) suggested individuals are less likely to provide care to others when they are multimorbid. Another study suggested multimorbidity is related to frailty among older caregivers (Alves et al., 2018). The restrictions in daily activities and additional health limitations due to multimorbidity might explain the connections. However, whether this results in social isolation remains unclear.

## Multiple Roles and Social Isolation Among the Middle-Aged Generation

A well-established aspect of middle age is the occupation of multiple work and familial roles. Recent studies have concentrated on family caregiving responsibility for adult family members, including children as well as aging parents and other relatives (Rozario et al., 2004), which have become common. For instance, about 35% of employed Canadians provide care to at least one family member or friend (The Vanier Institute of the Family, 2017), and about 17% of employed Canadians undertake the responsibility of childcare and eldercare simultaneously (Duxbury et al., 2009).

Although suggesting different consequences of holding multiple roles, both the role strain hypothesis and the role enhancement hypothesis have received substantial support from empirical studies. The role strain hypothesis proposes that the incompatible demands from different roles would make it stressful to manage inherent overload and conflict and lead to adverse health and wellbeing outcomes (Goode, 1960; Reid & Hardy, 1999). Studies have demonstrated higher anxiety levels and more depressive symptoms among individuals with multiple and cumulative role obligations (DeRigne & Ferrante, 2012; Kohl et al., 2018). Chassin et al. (2010) also showed that individuals with multiple roles, particularly those with caregiving responsibilities of older adults, adopt poor health behaviors since they tend to prioritize the health of other people.

The role enhancement hypothesis emphasizes the promoting function of complementary resources associated with different roles, which enable individuals to manage and balance multiple roles (Sieber, 1974). Role enhancement asserts that individuals can benefit from the resources, interaction, and recognition/reward from the multiple roles and the corresponding domains. Therefore, when individuals hold multiple roles, they tend to maintain considerable social ties and networks, to actively engage in a variety of domestic and social activities and events, as well as receive support from the network. A study based on women from both dual-earner and single-earner families (Sinha, 2017) found that employed women with dual roles reported better psychological wellbeing, and higher levels of life satisfaction. Another study found that among both women and men, participants who had three roles (i.e., working, childrearing, and caregiving to aging parents) had significantly less psychological distress than those who had fewer roles (Honda et al., 2015). Further, a recent longitudinal study also confirmed that caregivers who were in the paid labor force reported a lower level of loneliness than non-working caregivers (Ross et al., 2020). Workplace interaction and resources may provide a “protective shield” against the loneliness from family caregivers.

Thus, existent research on the influence of holding multiple roles on social isolation and well-being remains equivocal, and it appears that these associations are dependent on various conditions and/or moderating factors (Hansen & Slagsvold, 2015; Rozario et al., 2004). Some relevant factors are widely studied, such as gender or age, while some are not, including health conditions. For instance, significant gender differences were identified among the middle-aged generation in holding both family and work-related roles, including spouse, parent, worker, and caregiver (Kikuzawa, 2015), where a significant positive association between holding multiple roles and life satisfaction was identified among male participants, but not among their female counterparts. Age also makes a difference in understanding the impacts of having various roles. Younger and older mid-life individuals are at different developmental stages with disparities in personal responsibilities, resources, and physical health conditions (Carter et al., 2010). These life phase developmental contexts are all related to the capability of mid-life individuals to hold and balance demanding roles. Less is known about the influence of the multiple roles over time among individuals with different health conditions. One major reason is that health is usually examined as the outcome of holding several social and familial roles simultaneously, rather than a moderating effect (Barnett et al., 2012; Kohl et al., 2018; Li & Lee, 2020).

In summary, the middle-aged period entails a transition from young adult to older age, and it is a pivotal time in the life course (Duxbury et al., 2009). It is therefore critical to examine social isolation during this phase of life, considering its short- and long-term influence on wellbeing and health. The primary purposes of this study are to (1) examine the associations between three essential roles (parent, employee, and caregiver) and social isolation patterns among the middle-aged generation; and (2) further examine the interaction effects of multimorbidity by means of a comparative analysis across this major health condition during this important phase of the life course.

## Methods

This study employs a sub-sample of participants from the Canadian Longitudinal Study on Aging (CLSA). CLSA is a national wide long-term survey that started in 2010; and currently, two waves (∼3 years interval) of data have been collected from about 50,000 Canadians who are aged between 45 and 85 years old when recruited (51,338 participants for the baseline wave and 44,817 participants for the follow-up 1 wave). The CLSA data contain a wide range of information related to demographic background, health and wellbeing, family and social life, employment and retirement situation, etc. of participants (for detailed information, see Raina et al., 2009). The CLSA participants are comprised of two cohorts, the comprehensive cohort, and the tracking cohort. Participants from the comprehensive cohort were randomly selected from the population (within age/sex strata) within 25 km (50 km for lower population density areas) of the established 11 CLSA data collection sites, and data were collected through telephone interviews and an on-site face-to-face interview. Participants from the tracking cohort were randomly selected from the 10 provinces of Canada and data were collected through a telephone interview. The participation rate in the baseline wave of the study was 45% among eligible participants, and 10% overall (Raina et al., 2019). Data are available from the Canadian Longitudinal Study on Aging (www.clsa-elcv.ca) for researchers who meet the criteria for access to de-identified CLSA data.

This study involves 29,847 participants (14,777 participants with multimorbidity and 15,070 participants without multimorbidity) in the middle-age bracket between 45 and 64 years old at the baseline wave. Data from both baseline wave and follow-up 1 wave were included to conduct the longitudinal analysis.

### Measures

#### Social Isolation

Social isolation is a complicated and multifaceted concept with various definitions and measures. The current study follows the recent development of a multipronged definition of social isolation, which includes both objective structural/functional and subjective perceptive dimensions (Valtorta, Kanaan, Gilbody and Hanratty, 2016; Wister et al., 2019), rather than a single objective or subjective element. Social isolation was measured by the Social Isolation Index (SII) created by Wister et al. (2019) based on the CLSA Baseline data. The SII measure has established good concurrent validity, evidenced by its significant moderate association with several primary health and wellbeing outcomes (e.g., life satisfaction, depression). The SII is a composite index with three main dimensions: (1) structural/objective dimension: including community/social participation, size of social network, frequency of network contact, living arrangement, and marital status; (2) functional/objective dimension: including the four sub-scale of the Medical Outcome Study Social Support Survey Scale, emotional/informational support, affectionate support, tangible support, and positive social interaction; and (3) functional/subjective dimensions, including the loneliness evaluation item from the Center for Epidemiological Studies Depression Scale, and a perceptive question on the desire to engage in more social activities.

The standardized SII score ranges from 0 to 10, where 0 stands for least social isolation, and 10 means most socially isolated. In order to calculate the standardized SII score, all the sub-index variables from the three dimensions were summed and divided by the total score to transform them into a 0 to 10 standard sub-index. The scores of three dimensions were further summed and divided by three to calculate the SII score (for full details, see Wister et al., 2019). This study followed the same procedure and calculated the SII score for both baseline and follow-up 1 waves. See Supplemental Table 1 for detailed statistics of each of the SII variables at baseline and follow-up 1 waves, and among participants with and without multimorbidity, respectively.

#### Multiple Roles

Three roles were included in the data analysis, including employee role, parenting role, and caregiver role. Employee role was represented by the employment involvement level based on two questions, including “Are you currently working at a job or business?” and a follow-up question for those participants who were employed (or self-employed) “What is your current working status?.” The employment involvement variable was measured at three levels, including not employed, employed part-time, and full-time. The respondent's parenting role was assessed by the answers to the questions related to the number of people living with participants in the same household. Participants living with any child/minor under the age of 18 years old were viewed as parents with dependent minor(s). The number of dependent minor(s) was indicated at three levels, including no dependent minor, one dependent minor, and two or more dependent minors. Caregiving was captured by the survey question “During the past 12 months, have you provided any of the following types of assistance to another person because of a health condition or limitation?” Participants providing at least one type of assistance (e.g., personal care, medical care, housework, etc.) to other people were viewed as a family caregiver. Caregiving involvement was measured by the caregiving hours per week that participants provided to their main care receivers. In this study, caregiver involvement was measured at three levels, including not a caregiver, fewer than 5 h/week, and 5 h and more/week.

#### Multimorbidity

The CLSA invites participants to report 27 types of chronic conditions, including Alzheimer's disease, back problems, bowel incontinence, cancer, cataracts, diabetes, epilepsy, glaucoma, heart attack, heart disease, high blood pressure, irritable bowel syndrome, kidney disease, Parkinson's disease, peripheral vascular disease, lung disease, macular degeneration, multiple sclerosis, osteoarthritis, osteoporosis, migraine headaches, rheumatoid arthritis, stroke, thyroid problem, transient ischemic attack, ulcer, and urinary incontinence. Multimorbidity is defined as the occurrence of two or more of the 27 possible above-mentioned chronic conditions, and this variable is used for the separate comparative analyses to examine potential interaction effects of multiple roles and concurrent chronic illnesses. The status of multimorbidity or not was determined according to the baseline wave data.

#### Co-Variants

There are several demographic and socio-economic characteristics of participants that were controlled in the data analysis, including age, gender, highest educational attainment, annual household income, and ethnic background. Gender was collected as either male or female. Age was measured in actual years of old and was further recoded into two main age groups (45–54 and 55–64 years old). The highest education attainment was categorized into two levels, including less than post-secondary education, and post-secondary degree/diploma. Annual household income was measured at four levels, from <$50,000, $50,000–$99,999, $100,000–$149,999, till $150,000 or more. Ethnic background was indicated by whether participants belonged to a visible minority community or not.

In addition, two CLSA survey-related factors were included in the study. The first variable is the survey wave (baseline wave and follow-up 1 wave) and the second one, the survey type cohort (comprehensive and tracking cohorts). Thus, the survey wave and cohort variation were controlled in the data analysis.

### Analytic Procedure

Comparative bivariate analyses between participants with and without multimorbidity and related social-demographic factors are provided in Table 1. Comparative bivariate analyses between baseline wave and follow-up 1 wave regarding social isolation, and multiple role involvement, were further conducted to examine the changes over time between the two waves and shown in Table 2. In Table 3, the results of comparison between participants with and without multimorbidity regarding social isolation and multiple roles at both baseline wave and follow-up 1 wave are presented.

**Table 1.** Comparison Between Middle-Aged Participants With and Without Multimorbidity Regarding Social-Demographic Background.

Variables	All participants (*n* = 29,847)	Participants without multimorbidity (*n* = 14,777)	Participants with multimorbidity (*n* = 15,070)	*χ*^2^(*df*)/*t*-test
Cohorts				6.34 (1)*
Comprehensive	26.04	26.78	25.24	
Tracking	73.96	73.22	74.76	
Gender				449.52 (1)***
Male	49.41	54.66	43.69	
Female	50.59	45.34	56.31	
Age group				1545.90 (1)***
45–54 years old	54.88	64.12	44.83	
55–64 years old	45.12	35.88	55.17	
Highest education attainment				215.60 (1)***
Less than post-secondary education	24.09	20.64	27.85	
Post-secondary degree/diploma	75.91	79.36	72.15	
Household income				867.93 (3)***
<$50,000	20.39	14.69	26.65	
$50,000–$99,999	35.12	34.44	35.86	
$100,000–$149,999	23.57	25.97	20.93	
$150,000 or more	20.92	24.89	16.56	
Ethnic background				0.48 (1)
Visible minority	8.85	8.86	8.84	
Not visible minority	91.15	91.14	91.16	

*Note.* *p < .05; **p < .01; ***p < .001.

**Table 2.** Social Isolation and Multiple Roles Among Middle-Aged Participants From Baseline to Follow-Up 1.

Variables	Baseline	Follow-up 1	*χ*^2^(*df*)/*t*-test
	Mean (SD)/percentage		
Social isolation index score	3.02 (1.30)	3.07 (1.30)	−9.77***
Employment involvement			440.58(2)***
Not employed	33.00	39.18	
Employed part-time	11.74	14.26	
Employed full-time	55.26	46.56	
Amount of dependent minor			1092.53(2)***
No dependent minor	72.69	84.32	
One dependent minor	13.89	8.94	
Two and more dependent minors	13.42	6.74	
Caregiving hour per week			462.32(2)***
Not a caregiver	49.99	43.90	
Fewer than 5 h/week	39.23	43.77	
5 h and more/week	10.78	12.33	

*Note.* * *p* < .05; **p < .01; ***p < .001.

**Table 3.** Comparison Between Middle-Aged Participants With and Without Multimorbidity Regarding Social Isolation and Multiple Roles.

Variables	All participants (*n* = 29,847)	Participants without multimorbidity (*n* = 14,777)	Participants with multimorbidity (*n* = 15,070)	*χ*^2^(*df*)/*t*-test
Baseline wave				
Social isolation index score	3.02 (1.30)	2.93 (1.22)	3.13 (1.38)	−12.77***
Employment involvement				1407.81(2)***
Not employed	33.00	24.87	41.91	
Employed part-time	11.74	11.99	11.46	
Employed full-time	55.26	63.14	46.63	
Amount of dependent minor				923.38 (2)***
No dependent minor	72.69	66.57	79.35	
One dependent minor	13.89	15.91	11.70	
Two and more dependent minors	13.42	17.51	8.96	
Caregiving hour per week				91.35 (2)***
Not a caregiver	49.99	51.17	48.70	
Fewer than 5 h/week	39.23	39.35	39.10	
5 h and more/week	10.78	9.48	12.20	
Follow-up 1 wave				
Social isolation index score	3.07 (1.30)	2.98 (1.23)	3.16 (1.38)	−12.56***
Employment involvement				1313.66 (2)***
Not employed	39.18	30.79	48.56	
Employed part-time	14.26	14.31	14.20	
Employed full-time	46.56	54.90	37.24	
Amount of dependent minor				502.46 (2)***
No dependent minor	84.32	80.54	88.55	
One dependent minor	8.94	10.55	7.13	
Two and more dependent minors	6.74	8.90	4.32	
Caregiving hour per week				90.28 (2)***
Not a caregiver	43.90	44.68	43.03	
Fewer than 5 h/week	43.77	44.31	43.17	
5 h and more/week	12.33	11.01	13.81	

*Note.* *p < .05; **p < .01; ***p < .001.

Furthermore, linear mixed models (LMMs, Brown & Prescott, 2015) were applied in order to examine the longitudinal impact of multiple roles occupation on the social isolation reported by middle-aged participants with or without multimorbidity from baseline to follow-up 1 wave. LMM is featured with the merits to control the random effects due to repeated measures on the same individuals, and to incorporate both between-subject and within-subject variability in repeated measure studies (Fitzmaurice et al., 2012). In addition, LMM is advanced in conducting data analysis with both time-invariant factors (e.g., gender and education), and time-variant factors (e.g., role status and involvement in this study) in the same model.

Two hierarchical models were created to capture the association between multiple roles and social isolation over time. Model 1 included the basic survey factors and demographic and socio-economic variables. Model 2 modeled the relationship between involvement in the three key roles and social isolation among participants. To capture the relationship between multiple roles and the change of social isolation from baseline to follow-up 1 wave, interaction terms between survey wave and multiple roles variables were included in each model.

The model fit was estimated by the Akaike Information Criterion (AIC), and a lower number indicates a better model fit. In addition, according to CLSA (https://www.clsa-elcv.ca/), the trimmed weight was applied for descriptive analysis, and the analytic weight was applied for bivariate and multivariate analyses. For the SII score, missing values were replaced based on the imputed mode for categorical variables, median for ordinal variables, and mean for interval variables. All the independent variables have an acceptable number of missing cases (under 5%), and data analysis was conducted using the list-wise deletion method. Repeated analyses without imputed missing revealed replication of results, thus the imputed data are reported. The data were analyzed using SPSS Version 25.

## Results

A total of 29,847 participants were included in this study (see Table 1). The proportion of female participants was slightly higher than for males (51% vs. 49%). Approximately 55% of the participants were aged between 45 and 54 years old, and the majority of them have received post-secondary education (76%). The highest proportion of participants reported an annual household income from $50,000 to $99,999 (35%). Only a small proportion of participants identified as members of visible minority communities (9%).

The sample includes 14,777 participants who reported one or no chronic condition, and 15,070 participants reported two or more chronic conditions. After weighting, approximately 48% of the participants were multimorbid. Significant statistical differences exist between participants with and without multimorbidity based on: gender, age group, highest education, and household income. A higher proportion of female participants was multimorbid (56% vs. 45%), as well as those between 55 and 64 years old (55% vs. 36%). Also, a higher proportion of participants without multimorbidity received post-secondary degree or diploma (79% vs. 72%) and earned more than $100,000 yearly (51% vs. 37%).

The results regarding the changes of social isolation and the three studied roles (employee, parents, and caregiver) from baseline to follow-up 1 wave are shown in Table 2. Compared to the baseline wave, participants reported a higher score in SII at follow-up 1 wave (3.07 vs. 3.02, *p* < .001). At the baseline wave, about 67% of participants were employed (12% employed part-time and 55% employed full-time), and a significantly smaller proportion of participants (61%, 14% employed part-time, and 46% employed full-time, *p* < .001) were still employed at the follow-up 1 wave. The proportion of participants with dependent minor(s) at home was 27% at the baseline wave (14% with one dependent minor, and 13% with two or more dependent minors), and the number dropped to 16% at the follow-up 1 wave (9% with one dependent minor and 7% with two or more dependent minors). A different scenario is found with respect to the caregiving situation. A substantially higher proportion of participants were family caregivers at follow-up 1 wave (56% and 12% engaged in more than 5 h of caregiving weekly), compared to that of baseline wave (50% and 11% with more than 5 h weekly caregiving hour, *p* < .001).

Table 3 presents the group differences between participants with and without multimorbidity regarding SII, and the three roles at baseline wave and follow-up 1 wave, separately. Participants with multimorbidity reported higher scores of SII than those without multimorbidity at both baseline wave (3.13 vs. 2.93, *p* < .001), and follow-up 1 wave (3.16 vs. 2.98, *p* < .001). A higher proportion of participants without multimorbidity were employed when compared to participants with multimorbidity (75% vs. 58%, *p* < .001 at baseline, and 69% vs. 51%, *p* <.001 at follow-up 1). In addition, participants without multimorbidity were more likely to engage in a parenting role. A different situation was reported when comparing the caregiving role, where a slightly higher proportion of participants with multimorbidity identified themselves as family caregivers comparing to their counterparts without multimorbidity (51% vs. 49%, *p* < .001 at baseline, and 57% vs. 55%, *p* < .001 at follow-up 1). Also, slightly higher proportions of participants with multimorbidity provided more than 5 h of caregiving per week than those without multimorbidity at both baseline and follow-up 1 waves (12% vs. 9% and 14% vs. 11%, respectively).

Tables 4 and 5 show the results of LMM examining the associations between multiple roles occupation, key co-variants, and the social isolation of participants over time within participants without and with multimorbidity separately. Only Model 2 with all interested variables included was discussed here.

**Table 4.** Linear Mixed Models for Social Isolation Index Among Middle-Aged Participants Without Multimorbidity (*N* = 14,777).

Variables	Model 1		Model 2	
	Estimate	95% CI	Estimate	95% CI
Intercept	2.60***	2.55/2.66	2.63***	2.56/2.69
Survey wave				
Follow-up 1	0.03**	0.01/0.05	0.10***	0.06/0.14
Baseline (ref.)				
Cohorts				
Comprehensive	−0.02	−0.06/0.02	−0.01	−0.04/0.03
Tracking (ref.)				
Gender				
Male	0.08***	0.05/0.12	0.08***	0.05/0.12
Female (ref.)				
Age group				
45–54 years old	−0.08***	−0.11/−0.04	−0.01	−0.05/0.02
55–64 years old (ref.)				
Highest education attainment				
Less than post-secondary education	−0.02	−0.07/0.02	−0.04	−0.08/0.01
Post-secondary degree/diploma (ref.)				
Household income				
<$50,000	1.23***	1.18/1.29	1.21***	0.15/0.17
$50,000–$99,999	0.47***	0.42/0.51	0.45***	0.40/0.49
$100,000–$149,999	0.12***	0.07/0.16	0.11***	0.06/0.15
$150,000 or more (ref.)				
Ethnic background				
Visible minority	0.03	−0.03/0.09	0.04	−0.02/0.10
Not visible minority (ref.)				
Chronic conditions				
No condition	−0.03	−0.07/0.01	−0.02	−0.06/0.02
One condition (ref.)				
Chronic conditions × survey wave				
No condition × survey wave	0.03*	0.001/0.06	0.03	−0.001/0.06
One condition × survey wave (ref.)				
Employment involvement				
Employed part-time			−0.01	−0.06/0.04
Employed full-time			0.03	−0.005/0.07
Not employed (ref.)				
Amount of dependent minor				
One dependent minor			−0.16***	−0.21/−0.12
Two and more dependent minors			−0.25***	−0.29/−0.20
No dependent minor (ref.)				
Caregiving hour per week				
Fewer than 5 h/week			−0.03	−0.06/0.005
5 h and more/week			−0.04	−0.09/0.01
Not a caregiver (ref.)				
Employment involvement × survey wave				
Employed part-time × survey wave			−0.06	−0.12/0.001
Employed full-time × survey wave			−0.09***	−0.13/−0.04
Not employed × survey wave (ref.)				
Amount of dependent minor × survey wave				
One dependent minor × survey wave			−0.01	−0.06/0.05
Two and more dependent minors × survey wave			0.05	−0.003/0.10
No dependent minor × survey wave (ref.)				
Caregiving hour per week × survey wave				
Fewer than 5 h/week × survey wave			−0.07**	−0.11/−0.03
5 h and more/week × survey wave			−0.02	−0.09/0.05
Not a caregiver × survey wave (ref.)				
AIC	96607.19		94788.06	

*Note.* *p < .05; **p < .01; ***p < .001; The reference group in the analysis is indicated by (ref.).

**Table 5.** Linear Mixed Models for Social Isolation Index Among Middle-Aged Participants With Multimorbidity (*N* = 15,070).

Variables	Model 1		Model 2	
	Estimate	95% CI	Estimate	95% CI
Intercept	2.60***	2.53/2.66	2.64***	2.57/2.71
Survey wave				
Follow-up 1	0.08***	0.06/0.11	0.07***	0.03/0.11
Baseline (ref.)				
Cohorts				
Comprehensive	−0.03	−0.07/0.01	−0.03	−0.07/0.01
Tracking (ref.)				
Gender				
Male	0.08***	0.04/ 0.12	0.07***	0.03/0.11
Female (ref.)				
Age group				
45–54 years old	−0.03	−0.07/ 0.01	−0.003	−0.05/0.04
55–64 years old (ref.)				
Highest education attainment				
Less than post-secondary education	−0.03	−0.08/ 0.01	−0.04	−0.09/0.01
Post-secondary degree/diploma (ref.)				
Household income				
<$50,000	1.37***	1.31/1.43	1.36***	1.30/1.43
$50,000–$99,999	0.51***	0.45/0.57	0.51***	0.45/0.57
$100,000–$149,999	0.13***	0.06/0.19	0.13***	0.06/0.19
$150,000 or more (ref.)				
Ethnic background				
Visible minority	0.06	−0.01/0.13	0.07	−0.002/0.14
Not visible minority (ref.)				
Chronic conditions				
No condition	−0.06**	−0.10/–0.02	−0.07**	−0.11/−0.02
One condition (ref.)				
Chronic conditions × survey wave				
No condition × survey wave	−0.03	−0.07/0.01	−0.03	−0.06/0.01
One condition × survey wave (ref.)				
Employment involvement				
Employed part-time			0.01	−0.05/0.07
Employed full-time			0.06**	0.02/0.10
Not employed (ref.)				
Amount of dependent minor				
One dependent minor			−0.12***	−0.17/−0.06
Two and more dependent minors			−0.23***	−0.30/−0.16
No dependent minor (ref.)				
Caregiving hour per week				
Fewer than 5 h/week			−0.08***	−0.11/−0.04
5 h and more/week			−0.14***	−0.19/−0.08
Not a caregiver (ref.)				
Employment involvement × survey wave				
Employed part-time × survey wave			−0.03	−0.10/0.04
Employed full-time × survey wave			−0.01	−0.06/0.03
Not employed × survey wave (ref.)				
Amount of dependent minor × survey wave				
One dependent minor × survey wave			−0.10**	−0.18/−0.03
Two and more dependent minors × survey wave			−0.03	−0.12/0.05
No dependent minor × survey wave (ref.)				
Caregiving hour per week × survey wave				
Fewer than 5 h/week × survey wave			0.04	−0.01/0.08
5 h and more/week × survey wave			0.10**	0.03/0.16
Not a caregiver × survey wave (ref.)				
AIC	94928.68		92856.33	

*Note.* *p < .05; **p < .01; ***p < .001; The reference group in the analysis is indicated by (ref.).

The participants without multimorbidity reported a significant increase in SII score from baseline to follow-up 1 wave (estimate = 0.10, *p* < .001). Male participants reported higher social isolation than their female counterparts (estimate = 0.08, *p* < .001). When compared to participants with an annual household income over $150,000, those with an annual household income at lower levels all reported higher scores of SII (estimate ranges from 0.12 to 1.23, all *p* < .001). However, no statistically significant difference was identified based on participants’ gender, age group, education attainment, ethnical background, or number of chronic conditions (see Table 4).

The association between employment involvement and social isolation was not support, although the interaction between employment and survey wave was significantly related to social isolation. When compared to participants who were not employed, those who were employed full-time reported a significantly lower SII score over time (estimate = −0.09, *p* < .001). The SII scores among participants living with one dependent minor (estimate = −0.16, *p* < .001) and two or more dependent minors (estimate = −0.25, *p* < .001) reported lower levels of SII than those who were not living with any dependent minor. However, the association between social isolation and the interactive term between parenting role and survey wave is not supported. When it comes to caregiving role, the caregiving involvement was not statistically correlated with social isolation. Over the course of survey, participants with fewer than five caregiving hours per week reported significantly lower SII score comparing to those who were not caregivers (estimate = −0.07, *p* < .001).

Among participants with multimorbidity, the associations between social-demographic factors and SII scores reveal similar associations with that among participants without multimorbidity (see Table 5), except for a number of chronic conditions. Participants with two chronic conditions reported lower SII levels than those with three or more conditions (estimate = −0.07, *p* < .01), although a statistically significant difference was not identified over the course of survey. When compared to participants who were not employed, those who were employed full-time reported a significantly higher SII score (estimate = 0.06, *p* < .01). Both more dependent minors and the interactive terms between amount of dependent minors had statistically significant associations with social isolation. Participants living with one dependent minor reported lower SII scores than those who were not living with a dependent minor (estimate = −0.12, *p* < .001); and those with two or more dependent minors (estimate = −0.23, *p* < .001). Additionally, the increased rate of the SII score from baseline to follow-up 1 wave among participants living with one dependent minor is smaller than that of participants who had no dependent minor in the household (estimate = −0.10, *p* < .01). Interestingly, caregivers providing fewer than 5 h per week reported lower SII scores than non-caregivers (estimate = −0.08, *p* < .001); as well as those giving 5 or more hours per week (estimate = −0.14, *p* < .001). Caregivers with higher caregiving intensity (5 h and more/week) experienced a greater increase in SII score than non-caregivers from baseline to follow-up 1 wave (estimate = 0.10, *p* < .01).

## Discussion

Parenting, eldercare, and work roles have become more fluid and complex during the middle phases of life and can have negative consequences for social isolation experiences. These roles are further nuanced by the presence of multimorbidity, which often manifests during the mid-life stages of development. The current study seeks to understand social isolation among middle-aged persons with a focus on the influence of holding multiple familial and work roles over time, and comparatively, among individuals with and without multimorbidity. Indeed, our research supports and extends a life course theoretical perspective by finding that: (1) the middle-aged generation experiences slightly greater social isolation over time; (2) middle-aged individuals without multimorbidity, who hold multiple roles (parenting dependent minors, employment, and caregiving), have lower social isolation; and (3) middle-aged individuals with multimorbidity, who are fully employed and who have higher caregiving intensity (5 h or more per week) have higher social isolation.

Furthermore, middle-aged participants (with or without multimorbidity) reported a slightly higher level of social isolation at the follow-up 1 than during the baseline wave, which is approximately 3 years apart. Although the effect size is small, this finding is consistent with previous studies suggesting that as people age, they tend to experience social isolation due to shrinking social network size, participation in fewer social and leisure activities, and also greater risk of feelings of loneliness (Thomas, 2011). Based on a meta-analysis with 277 studies, Wrzus et al. (2013) concluded that an individual's network size increases during adolescence and young adulthood keeps stable in the 20 and 30 s, and then starts a continuous decline from adulthood to older age. Furthermore, in a recent study based on community-dwelling adults in the United States (Lee et al., 2019), there were three life stages at which point participants reported the severest loneliness level, including young adulthood (late-20 s), middle-age (mid-50 s), and old-old age (late-80 s).

Our study observed changes in the three studied roles from baseline to follow-up 1 wave, reflecting pivotal life course transitional shifts between the middle-aged to older age periods, and aligning with other studies on these developmental stages (e.g., Lachman et al., 2015). As expected, over time, engagement in parenting dependent minor(s) and employment decrease with age. It is normal that during the middle-aged period, children mature into young adults and tend to be more independent, including leaving home for education or jobs, settling into careers, and forming their own households and families (Mitchell, 2007). Therefore, from the middle to older age period, parents begin to experience the transition from empty nest to family dissolution. Indeed, the empty nest is a widely studied topic among the middle to old age population, and the permanent departure of children has a fundamental impact on family transitions and the daily lives of parents (Mitchell, 2019; Mitchell & Lovegreen, 2009; Mitchell et al., 2020).

Furthermore, after entering the middle-aged developmental phase, particularly during the late 50 s, individuals tend to gradually release from their social roles, such as employment. According to Statistics Canada (2020), the employment rate among different age groups peaks at the age group of 40–44 years old (84.7%), followed by gradual declines from 45 years to the older groups (83.4% for 45–49 years, 81.3% for 50–54 years, 71.8% for 55–59 years, 53.1% for 60–64 years, and 25.3% for 65–69 years, respectively). A similar situation was reported among OECD countries (OECD, 2017), showing that the working hours per week among individuals aged 50 years and older decreased gradually, and the employment rate fell sharply among individuals aged in the 50 and 60 s. The employment situation of participants in this study is consistent with the identified trend in Canada and other developed countries.

Although more intensive involvement in parenting dependent minor(s) and employment reduces over time, the middle-aged generation often faces increased responsibility in providing care and help to family members (i.e., aging parents, spouses) or friends. According to Fast et al. (2013), the onset of eldercare in Canada occurs right after entering the middle-aged period (around 46–48 years old). Anderson et al. (2013) also suggested that individuals aged between 50 and 64 years old comprise a significantly higher proportion of caregivers than other younger and older age groups. In our study, the proportion of participants who viewed themselves as family caregivers increased from 50 to 56% in roughly 3 years.

The findings regarding the relationship between occupying multiple roles and social isolation among participants without multimorbidity over time support the enhancement hypothesis. However, the findings yielded from participants with multimorbidity regarding the long-term impact of different roles are mixed, revealing evidence consistent with both role enhancement and role strain perspectives based on the direction of the associations. This further confirms the necessity to consider multimodality as a salient contextual factor in examining the impact of holding multiple roles.

Among participants without multimorbidity, all three studied roles are negatively associated with social isolation either concurrently or longitudinally. Seminal studies (Moen et al., 1992; Thoits, 1983) have suggested that being engaged in multiple roles reflects the maintenance of social identities/positions, as well as continuity in social relationships associated with a series of activities and/or responsibilities. Multiple roles appear to particularly critical during the middle to later life, considering that individuals tend to lose roles rather than accumulate roles during aging, and that role loss is a significant factor contributing to social isolation (Keefe et al., 2006). For instance, a recent literature review (Cotterell et al., 2018) suggested that during the life-course transition from middle to later life, risk factors for social isolation include retirement, loss of income, and having children leave the family or relocate a long-distance away. The findings in our study regarding the associations between being employed full-time, parenting dependent children, and moderate caregiving responsibility and social isolation among participants without multimorbidity resonate with previous literature. However, this does not preclude the co-occurrence of negative consequences associated with multiple roles, such as stress and burden, in tandem with positive ones.

In our study, parenting-dependent minors are associated with lower levels of social isolation among both groups of middle-aged participants with and without multimorbidity. There are several reasons to explain this finding. Typically, as children grow older transitioning from elementary school to high school, parental involvement in children's learning, school activities, and extracurricular activities gradually declines over time (Crosnoe, 2001). Attending school activities and other events promotes the opportunity for social participation with teachers and other parents. Another potential reason for developmental patterns and roles affecting social isolation is related to the empty nest syndrome. Researchers have also suggested that when children leave home, middle-aged parents may experience loneliness due to identity and role loss (Mitchell & Lovegreen, 2009). Mitchell and Wister (2015) also found that some middle-aged parents view children leaving home as a process of losing companionship, as well as affective and instrumental support. Thus, some parents feel greater social isolation after children grow to be independent and leave home. Additionally, one early study on the empty nest syndrome reported that the positive effects of empty nest disappeared after 2 years (Harkins, 1978). However, the association between less involvement in parenting minor children and social isolation tended to be stable over 3 years in our study.

Additionally, among participants without multimorbidity, we found that participants who were employed full-time had a slower increase in social isolation over time compared to those who were unemployed from baseline to follow-up 1 wave. Employment is one of the most essential social roles that individuals hold contributing to work-based identity, financial resources, and social network and social connectedness (Holt-Lunstad, 2017). Compared to those who are employed, unemployed individuals are less likely to actively participate in club/organization events, to go out, to contact friends, or even to seek help from others (Dieckhoff & Gash, 2014). Unemployment is associated with adverse short- and long-term physical and psychological health, and opportunity for re-employment (Krueger et al., 2014).

Conversely, among participants with multimorbidity, those who were employed full-time experienced a greater increase in social isolation than those who were not employed (but not between participants who were unemployed and employed part-time). Previous studies have pointed out that individuals with multimorbidity are more likely to transfer to part-time employment or full retirement (van Zon et al., 2020). In this study, the fact that around half of the participants with multimorbidity were unemployed at both baseline and follow-up 1 waves is consistent with previous findings. Yet, previous evidence has also established the connection between multimorbidity and high personal expenditures on medicines (Sum et al., 2018). Financial strain may require that individuals with multimorbidity need to work full-time. As a result, this group may find it challenging to manage full-time employment and other aspects of life, and thus sacrifice their social life. Conversely, those individuals with multimorbidity who reduce employment may be able to shift focus on to family oriented or friendship relationships. Indeed, studies have identified the connection between multimorbidity and increased network size, including family and friends, as well as service providers and health professionals (Kristensen et al., 2019; Mckinlay et al., 2017). The enlarged network size might be related to a greater need for support in daily life and health care among individuals with multimorbidity. These positive consequences might offset feelings of loneliness and exclusion (Kristensen et al., 2019). However, clearly more studies are needed to further examine the influence of multimorbidity on social isolation, both comprehensively and dimensionally.

In this study, caregiving role functions differentially among participants with and without multimorbidity. Among participants without multimorbidity, being a caregiver with moderate caregiving intensity (fewer than 5 h/week) over time is associated with lower social isolation. This finding is consistent with previous research that has identified positive social aspects of caregiving, such as maintaining contact with, and receiving more support, from family members, as well as enhancing relationships between caregivers and receivers or other family members (Hango, 2020). However, when participants with multimorbidity act as family caregivers and provide intensive caregiving (5 h or more/week) for a considerable duration, they tend to experience a greater increase in social isolation over time compared to non-caregivers. This finding is consistent with previous literature, in that long-term intensive caregiving tends to reduce social contact, narrow the social network, lower employment involvement or result in premature retirement, and leads to feeling lonely and hopeless (Li et al., 2020; Poon et al., 2017).

These unique relationships between caregiver role and social isolation among participants with or without multimorbidity further emphasize the importance of caregiver health condition in managing caregiving tasks with other work and life responsibilities. Family caregivers with compromised health tend to find it difficult to manage caregiving tasks, and conversely, physical health may suffer due to being a family caregiver, although the latter direction was not investigated in our study. Indeed, we found a higher proportion of participants with multimorbidity who were family caregivers, and provided more intensive caregiving than those without multimorbidity. This creates a double jeopardy for participants with multimorbidity, since they need to take care of themselves and another family member or members concurrently.

This study also supports the significant association between several socio-demographic factors and social isolation among the middle-aged generation. Men reported greater social isolation over time than women. Previous evidence on the relationship between gender and social isolation has been inconsistent (Gilmour & Ramage-Morin, 2020; Vandervoort, 2000). One potential explanation of the gender difference is that men tend to have the smaller and less stable social networks in mid-life (Klinenberg, 2016). Men tend to receive emotional support from their spouses, while women obtain support from a broader network (Vandervoort, 2000). Household income is also significantly related to social isolation. It is understandable that individuals and families with higher income tend to have more social and financial resources to manage complicated roles, and are better able to keep connected with networks (e.g., Gilmour & Ramage-Morin, 2020). Additionally, the level of multimorbidity was associated with social isolation only when participants have three or more conditions compared to only two conditions. This finding further implies the need for future studies to compare different measures of multimorbidity (Harrison et al., 2014; Wister et al., 2015).

### Implications

Midlife is a complex and pivotal life course developmental phase at which time social and work roles, coupled with multimorbidity, are concurrently and dynamically related to social isolation. The present study suggests that as the middle-aged population ages, they are more likely to experience social isolation, albeit the change is small in this study. Our research findings also support both role enhancement and role strain perspectives. Ideally, we need to conduct additional longitudinal studies in order to examine the fluidity of multiple roles circumstances across the life cycle, rather than as static stages or statuses, as well as expanding this work to examine several outcomes simultaneously.

Our findings regarding the negative associations between employment, caregiving and social isolation among participants with multimorbidity also call for enhanced access to formal and informal support aimed at supporting mid-life adults with challenging circumstances. Individuals with multimorbidity tend to have more out-of-pocket expenditure on medicines, and the financial burden may further magnify the risk of social isolation. Policymakers and healthcare system reform need to consider different financial options for individuals with multimorbidity and provide wider health coverage such as pharma care, as well as health promotion (Wister et al., 2016). Also, people usually become family caregivers during the middle-aged period, and this caregiving role tends to continue due to the need to support aging parents, followed by spouses, siblings, or significant others during their own aging process. Therefore, strengthening informal support from other family members, and formal support from the community and health care systems, is needed. Hopefully, this support will help to prevent middle-aged individuals with multimorbidity from being overwhelmed and negatively affected by family caregiving responsibilities.

There are several limitations related to study design and sampling worthy of further consideration. The participation rate (45%) and overall response rate (10%) in the CLSA constrain the generalizability of the study; however, these non-response rates are similar to other major national cohort studies of aging populations (Raina et al., 2019). In addition, while the CLSA sample has a higher socio-economic status distribution than the Canadian population, the estimates of the associations based on multivariate analysis would not be significantly affected. Furthermore, the current study followed the recent development of conceptualizing social isolation with both objective and subjective components, and thus used the composite SII to measure social isolation (Wister et al., 2019). Within the sub-index, the marital status and living arrangement may have a confounding impact on the multiple roles held by participants. The study conducted data analyses with the adjusted SII (without marital status and living arrangement), and the results were replicated. Therefore, this study reported the results based on the original SII and enabled future comparison with other studies using the same index.

Further research is needed using different measures of social isolation, as well as different sub-populations and countries to reinforce and elucidate these findings.

Moreover, from the three essential roles studied, we chose to focus on parenting minor dependents under 18 years old in this study, rather than including all the children living in the same household. There exists an extensive proportion of literature focusing on parents living with adult children, and the nature of this co-residence is different but more complicated than parenting minor dependents (Mitchell, 2007). For instance, continued parenting roles past the age of 18 years old typically extend beyond raising and nurturing children, and is often associated with different financial and emotional support concerns, which in turn can create unique types of intergenerational conflict (Burn & Szoeke, 2016). Therefore, the relationship between continued parenting and social isolation might create a different set of stressors, responsibilities, and implications for social connectedness (i.e., social isolation) compared to parenting minor dependents. Finally, implications of this work should be made with caution when applying the findings to the broader population, since the effect sizes uncovered are extremely small. And it is therefore unclear whether they would have any clinical relevance, especially for changes in social isolation over time.

## Conclusion

This study focused on the middle-aged generation undertaking key social and familial roles, including parenting minor dependents, employment and family caregiving. Findings reveal associations between holding multiple roles and lower social isolation among middle-age generation without multimorbidity; but conversely, it also establishes the relationship between employment, caregiving and greater social isolation among middle-age generation with multimorbidity. The results yielded from this study provide evidence to advocate for service and programs tailored to support middle-aged family caregivers while also facing multimorbidity. In sum, this research raises important awareness and a number of salient issues underlying mid-life transitions. In particular, this novel research emphasizes the need to better understand the implications of complex, competing, and changeable role and status transitions in midlife and the value of adopting a strong life course gerontological lens.

## Supplementary Material

Supplementary material
