# Supplementary material for: A Study of Social Isolation, Multimorbidity and Multiple Role Demands Among Middle-Age Adults Based on the Canadian Longitudinal Study on Aging
Source: Int J Aging Hum Dev. 2021 Oct 12;94(3):312–43. doi: 10.1177/00914150211040451 (PMC8866749; doi:10.1177/00914150211040451)
Supplement: Supplementary material [file sj-pdf-1-ahd-10.1177_00914150211040451.pdf]

Supplementary table 1: Descriptive statistics for Social Isolation Index variables among middle-aged population

	Baseline			Follow-up 1		
	All participants	Participants without multimorbidity	Participants with multimorbidity	All participants	Participants without multimorbidity	Participants with multimorbidity
Ordinal Structural Objective Items: Community participation						
Frequency of participation in family/ friends activities						
At least once a day (0)	4.14	4.23	4.05	3.33	3.35	3.32
At least once a week (2.5)	47.32	49.44	44.93	45.06	45.28	44.91
At least once a month (5)	38.92	38.17	39.75	39.90	40.24	39.65
At least once a year (7.5)	8.57	7.37	9.91	10.01	10.06	9.97
Never (10)	1.05	.78	1.35	1.70	1.08	2.15
Frequency of participation in religious activities						
At least once a day (0)	.81	.73	.91	.49	.45	.52
At least once a week (2.5)	15.93	16.30	15.51	15.80	15.33	16.14
At least once a month (5)	9.37	9.33	9.40	9.06	9.28	8.91
At least once a year (7.5)	20.76	21.72	19.70	22.81	24.01	21.96
Never (10)	53.13	51.92	54.47	51.83	50.93	52.47
Frequency of participation in sports or physical activities						
At least once a day (0)	7.88	8.22	7.51	6.23	6.58	5.99
At least once a week (2.5)	44.97	48.68	40.80	43.68	47.59	40.89
At least once a month (5)	17.20	18.02	16.29	19.61	20.28	19.13
At least once a year (7.5)	7.64	7.50	7.80	8.23	7.88	8.48
Never (10)	22.31	17.58	27.60	22.25	17.68	25.50
Frequency of participation in educational or cultural activities						
At least once a day (0)	.99	1.05	.92	.60	.56	.63
At least once a week (2.5)	9.16	9.91	8.33	8.37	8.28	8.43
At least once a month (5)	40.91	43.27	38.27	38.85	41.57	36.91
At least once a year (7.5)	34.15	34.35	33.94	37.85	38.18	37.61
Never (10)	14.78	11.42	18.55	14.34	11.41	16.42
Frequency of participation in clubs or fraternal organization activities						
At least once a day (0)	.17	.13	.23	.13	.12	.15
At least once a week (2.5)	4.12	4.27	3.96	3.81	3.33	4.15
At least once a month (5)	8.32	8.03	8.65	8.96	8.16	9.53

At least once a year (7.5)	8.65	9.05	8.19	9.43	10.13	8.92
Never (10)	78.73	78.52	78.97	77.67	78.26	77.25
Frequency of participation in association activities						
At least once a day (0)	.63	.50	.78	.36	.35	.38
At least once a week (2.5)	6.29	6.61	5.93	5.03	4.87	5.15
At least once a month (5)	18.72	19.59	17.75	17.10	17.29	16.98
At least once a year (7.5)	24.37	25.58	23.02	27.54	30.68	25.29
Never (10)	49.98	47.72	52.51	49.96	46.82	52.21
Frequency of participation in volunteer or charity work						
At least once a day (0)	1.49	1.41	1.59	1.47	1.22	1.64
At least once a week (2.5)	13.16	13.35	12.94	14.34	13.10	15.23
At least once a month (5)	19.47	19.85	19.04	18.88	19.38	18.52
At least once a year (7.5)	23.57	25.18	21.76	25.26	27.72	23.51
Never (10)	42.32	40.21	44.67	40.06	38.58	41.11
Frequency of participation in other recreational activities						
At least once a day (0)	2.79	2.37	3.25	2.15	1.89	2.34
At least once a week (2.5)	24.99	25.10	24.88	22.63	21.45	23.47
At least once a month (5)	28.26	28.74	27.74	29.39	29.04	29.63
At least once a year (7.5)	12.59	13.07	12.06	14.47	15.38	13.83
Never (10)	31.37	30.73	32.09	31.35	32.24	30.72
Continuous structural objective items: Transformed social network quantity						
Number of children (0 to 10)	8.94 (0.73)	8.94 (0.72)	8.93 (0.73)	8.77 (0.84)	8.76 (0.83)	8.77 (0.84)
Number of siblings (0 to 10)	8.73 (0.94)	8.75 (0.92)	8.71 (0.97)	8.77 (0.83)	8.89 (0.79)	8.85 (0.87)
Number of relatives (0 to 10)	6.15 (2.98)	6.19 (2.92)	6.10 (3.03)	6.21 (2.79)	6.21 (2.73)	6.21 (2.84)
Number of friends (0 to 10)	9.37 (0.75)	9.34 (0.79)	9.40 (0.71)	9.40 (0.66)	9.36 (0.72)	9.42 (0.62)
Number of neighbours (0 to 10)	8.83 (1.45)	8.82 (1.43)	8.84 (1.47)	8.88 (1.35)	8.88 (1.33)	8.88 (1.36)
Number of people from work/school (0 to 10)	4.09 (3.90)	3.84 (3.79)	4.38 (4.00)	4.76 (3.93)	4.43 (3.81)	4.99 (4.00)
Number of people from community (0 to 10)	6.55 (3.75)	6.44 (3.77)	6.68 (3.73)	6.87 (3.55)	6.81 (3.57)	6.91 (3.54)
Number of people from other activity (0 to 10)	7.61 (3.28)	7.50 (3.33)	7.74 (3.22)	7.74 (3.02)	7.69 (3.03)	7.78 (3.02)
Ordinal structural objective items: Last time visited						
Last time visited: Children outside of household						
Within the last day or two (0)	20.72	18.62	23.07	23.35	21.23	24.86
Within the last week or two (2)	24.08	22.42	25.95	29.35	28.77	29.76
Within the past month (4)	7.34	6.91	7.83	8.52	8.16	8.77
Within the past 6 months (6)	5.98	5.47	6.54	6.46	6.43	6.49
Within the past year (8)	.93	.85	1.03	.95	.81	1.05
More than 1 year ago (10)	40.95	45.74	35.58	31.37	34.61	29.06

Last time visited: Siblings outside of household						
Within the last day or two (0)	11.49	10.98	12.06	11.51	10.81	12.02
Within the last week or two (2)	26.50	26.79	26.19	25.01	25.37	24.76
Within the past month (4)	17.13	17.71	16.48	17.68	18.45	17.13
Within the past 6 months (6)	23.43	24.12	22.66	23.54	24.09	23.14
Within the past year (8)	5.95	5.95	5.94	6.30	6.56	6.12
More than 1 year ago (10)	15.50	14.45	16.68	15.96	14.72	16.84
Last time visited: Other relatives outside of household						
Within the last day or two (0)	13.60	12.90	14.39	16.51	15.31	17.37
Within the last week or two (2)	25.10	25.25	24.94	30.22	31.14	29.56
Within the past month (4)	17.12	17.61	16.56	17.65	17.86	17.50
Within the past 6 months (6)	22.46	22.84	22.03	19.20	19.62	18.90
Within the past year (8)	7.48	7.70	7.25	6.48	6.73	6.30
More than 1 year ago (10)	14.24	13.70	14.84	9.94	9.35	10.36
Last time visited: Close friends outside of household						
Within the last day or two (0)	31.16	30.44	31.96	29.45	28.41	30.20
Within the last week or two (2)	40.41	41.15	39.58	42.49	44.59	40.99
Within the past month (4)	11.94	12.29	11.54	12.79	13.13	12.54
Within the past 6 months (6)	8.28	8.52	8.02	7.47	7.33	7.57
Within the past year (8)	1.43	1.45	1.41	1.40	1.11	1.60
More than 1 year ago (10)	6.78	6.16	7.48	6.40	5.42	7.09
Last time visited: Neighbours outside of household						
Within the last day or two (0)	21.46	20.70	22.31	24.87	23.40	25.92
Within the last week or two (2)	30.99	30.95	31.04	33.90	35.45	32.80
Within the past month (4)	10.39	10.79	9.94	10.58	10.46	10.66
Within the past 6 months (6)	11.25	11.72	10.72	10.47	10.95	10.13
Within the past year (8)	3.57	3.44	3.71	3.51	3.31	3.66
More than 1 year ago (10)	22.34	22.40	22.27	16.66	16.43	16.83
Ordinal structural objective weighted item						
Living alone						
No (0)	87.73	89.73	85.49	85.96	88.72	84.00
Yes (10)	12.27	10.27	14.51	14.04	11.28	16.00
Marital status						
Partnered (0)	77.74	80.23	74.96	78.15	81.59	75.70
Not partnered (10)	22.26	19.77	25.04	21.85	18.41	24.30
Continuous functional objective items: Transformed MOS social support scale						
Affectional support (0 to 10)	1.22 (1.90)	1.08 (1.75)	1.37 (2.05)	1.22 (1.93)	1.07 (1.75)	1.33 (2.04)
Emotional/Informational support (0 to 10)	1.74 (1.85)	1.60 (1.72)	1.92 (1.98)	1.67 (1.85)	1.48 (1.68)	1.81 (1.95)

Positive Social Interaction (0 to 10)	1.59 (1.89)	1.45 (1.75)	1.76 (2.03)	1.52 (1.87)	1.34 (1.72)	1.64 (1.97)
Tangible support (0 to 10)	1.77 (2.03)	1.64 (1.89)	1.93 (2.16)	1.67 (2.02)	1.54 (1.88)	1.77 (2.10)
Ordinal functional subjective items						
How often did you feel lonely?						
All the time (10)	78.01	80.88	74.80	79.39	83.69	76.34
Occasionally (6.67)	12.15	11.22	13.18	11.39	9.48	12.75
Some of the time (3.33)	7.45	6.42	8.59	6.79	5.35	7.82
Rarely or never (0)	2.40	1.47	3.43	2.42	1.48	3.09
Desire to participate in more activities						
No (10)	48.15	46.79	49.67	43.63	41.89	44.87
Yes (0)	51.85	53.21	50.33	56.37	58.11	55.13
